# ATP-Induced Increase in Intracellular Calcium Levels and Subsequent Activation of mTOR as Regulators of Skeletal Muscle Hypertrophy

**DOI:** 10.3390/ijms19092804

**Published:** 2018-09-18

**Authors:** Naoki Ito, Urs T. Ruegg, Shin’ichi Takeda

**Affiliations:** 1 Department of Molecular Therapy, National Institute of Neuroscience, National Center of Neurology and Psychiatry, Kodaira 187-8502, Japan; 2 Pharmacology, Geneva-Lausanne School of Pharmaceutical Sciences, University of Geneva, CH 1211 Geneva, Switzerland; urs.ruegg@unige.ch

**Keywords:** skeletal muscle, muscle hypertrophy, muscle atrophy, ATP, Ca^2+^, P2Y receptor, IP_3_ receptor, mammalian target of rapamycin (mTOR), mitogen-activated protein kinase (MAPK)

## Abstract

Intracellular signaling pathways, including the mammalian target of rapamycin (mTOR) and the mitogen-activated protein kinase (MAPK) pathway, are activated by exercise, and promote skeletal muscle hypertrophy. However, the mechanisms by which these pathways are activated by physiological stimulation are not fully understood. Here we show that extracellular ATP activates these pathways by increasing intracellular Ca^2+^ levels ([Ca^2+^]_i_), and promotes muscle hypertrophy. [Ca^2+^]_i_ in skeletal muscle was transiently increased after exercise. Treatment with ATP induced the increase in [Ca^2+^]_i_ through the P2Y_2_ receptor/inositol 1,4,5-trisphosphate receptor pathway, and subsequent activation of mTOR in vitro. In addition, the ATP-induced increase in [Ca^2+^]_i_ coordinately activated Erk1/2, p38 MAPK and mTOR that upregulated translation of JunB and interleukin-6. ATP also induced an increase in [Ca^2+^]_i_ in isolated soleus muscle fibers, but not in extensor digitorum longus muscle fibers. Furthermore, administration of ATP led to muscle hypertrophy in an mTOR- and Ca^2+^-dependent manner in soleus, but not in plantaris muscle, suggesting that ATP specifically regulated [Ca^2+^]_i_ in slow muscles. These findings suggest that ATP and [Ca^2+^]_i_ are important mediators that convert mechanical stimulation into the activation of intracellular signaling pathways, and point to the P2Y receptor as a therapeutic target for treating muscle atrophy.

## 1. Introduction

Skeletal muscle maintains an adequate volume that is commensurate with its surrounding environment. This is regulated by a balance between protein synthesis and degradation [1]. The insulin-like growth factor-1 (IGF-1) activates the mammalian target of rapamycin (mTOR) through the class I phosphatidylinositol 3-kinase (PI3K)/Akt pathway, which induces protein synthesis and subsequent muscle growth or hypertrophy [2]. Physiological stimulation, such as exercise and mechanical load, also promotes muscle hypertrophy by upregulating protein synthesis [3]. However, recent studies have revealed that exercise- or mechanical load-induced activation of mTOR is not mediated by the IGF-1/class I PI3K/Akt pathway, especially at an early stage of muscle hypertrophy [4,5,6,7]. This suggests the presence of another mediator that converts physiological stimulation into the activation of mTOR. We have previously demonstrated that mechanical load-induced activation of mTOR and subsequent muscle hypertrophy were regulated by a TRPV1-mediated increase in intracellular Ca^2+^ levels ([Ca^2+^]_i_) [8,9]. However, it is not clear whether TRPV1 is the only channel that converts mechanical load into intracellular signaling events that induce muscle hypertrophy. This motivated us to investigate the potential role of other Ca^2+^ channels involved in this process.

Release of ATP from muscles in response to contraction, exercise or electrical stimulation was observed both in humans and in rodents [10,11,12,13]. ATP and other purine- or pyrimidine-based agonists mediate a variety of biological functions by activating purinergic receptors, namely the ligand-gated P2X receptors or the G-protein-coupled P2Y receptors [14]. Activation of P2Y receptors induces the production of inositol 1,4,5-trisphosphate (IP_3_) through the Gαq–mediated activation of phospholipase C (PLC), thereby causing Ca^2+^ to be released from the sarcoplasmic reticulum (SR)-localized inositol 1,4,5-trisphosphate receptors (IP_3_R) [15,16]. However, to our knowledge, the role of ATP in the activation of mTOR and muscle hypertrophy has not been analyzed

## 2. Results

### 2.1. ATP Induces an Increase in [Ca^2+^]_i_ by Activating the P2Y_2_ Receptor and Downstream IP_3_R

**Figure 1:**
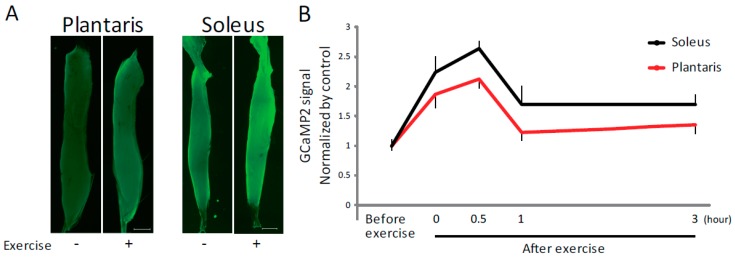
Exercise induces increased intracellular Ca^2+^ levels. (**A**) Representative fluorescence imaging of isolated plantaris and soleus muscles from pCAGGS-GCaMP2 mice before and after exercise. Scale bar: 1 mm. (**B**) Quantitative analysis of GCaMP2 signal intensity (*n* > 4). Plantaris and soleus muscles were isolated before or immediately, 0.5, 1 and 3 h after exercise. Error bars indicate S.E.M.

Plasma ATP levels are known to increase following exercise or muscle contraction [10,11,12,13]. To investigate the functional consequences of this increase, we focused on ATP in regulating [Ca^2+^]_i_ and subsequent muscle hypertrophy. Using pCAGGS-GCaMP2 mice, which express a genetically encoded Ca^2+^ indicator [17], we found that [Ca^2+^]_i_ increased after exercise in both the soleus and plantaris muscles, which are mainly composed of slow and fast muscle fibers, respectively (Figure 1A). This increase was observed immediately after and during 30 min after exercise (Figure 1B), suggesting a causal relationship between exercise-induced increase of plasma ATP levels and exercise-induced increase in [Ca^2+^]_i_.

**Figure 2:**
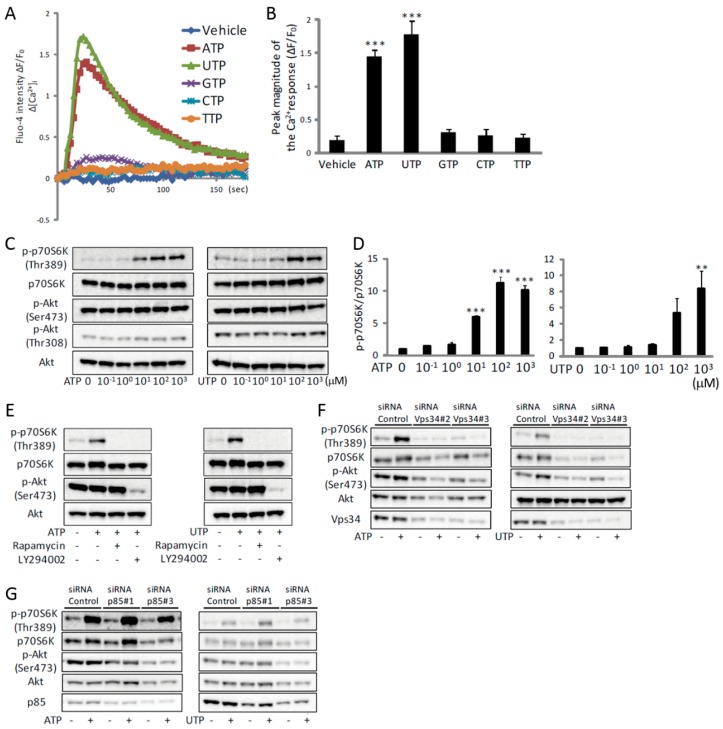
ATP induces an increase in [Ca^2+^]_i_ and activation of mTOR via Vps34 in C2C12 myotubes. (**A**) Traces of Fluo-4 fluorescence in C2C12 myotubes treated with nucleotide triphosphates at a concentration of 100 µM (*n* = 4). (**B**) Quantitative analysis of peak magnitudes of (**A**) (*n* = 4). (**C**) Representative Western blot analysis showing concentration-dependent effects of ATP (left) or UTP (right) on phosphorylation of p70S6K and Akt (*n* = 4). (**D**) Quantitative signal intensities of p-p70S6K/p70S6K in (**C**) (*n* = 4). (**E**–**G**) Representative Western blot analysis showing the effects of rapamycin or LY294002 (E), siRNA for Vps34 (**F**) or p85 (**G**) on ATP- or UTP-induced phosphorylation of p70S6K and Akt (*n* = 4). The concentration of ATP and UTP in (**E**–**G**) was 100 µM and the duration of treatment in all experiments shown in this figure was 30 min. ** *p* < 0.01, *** *p* < 0.001 by ANOVA with Tukey-Kramer test. Error bars indicate S.E.M.

We next analyzed the ATP-induced Ca^2+^ signaling events in vitro. Similarly to previous reports [18,19,20,21,22,23,24], exogenous ATP treatment of C2C12 myotubes resulted in a transient increase in [Ca^2+^]_i_ (Figure 2A,B). This increase was also observed by the closely related pyrimidine triphosphate UTP, whereas GTP, CTP and TTP had no effect (Figure 2A,B). ATPγS or 2-MeS ATP also increased [Ca^2+^]_i_, whereas ADP, AMP or adenosine had no effect (Supplementary Figure S1A). ATP or UTP induced an increase in [Ca^2+^]_i_ in a concentration-dependent manner (Supplementary Figure S1B,C). To investigate which receptor was mediating the ATP- or UTP-induced increase in [Ca^2+^]_i_, we examined P2Y receptors and the subsequent PLC-mediated activation of IP_3_R. Using mouse NG108-15 cells, Lustig et al. showed that the relative agonist potencies on the P2Y_2_ receptor were ATP = UTP > ATPγS > 2-MeS ATP [25]. Our similar results suggest that the P2Y_2_ receptor may be the target of ATP in C2C12 myotubes (Supplementary Figure S1A). In agreement with this, the ATP- or UTP-induced increase in [Ca^2+^]_i_ was prevented either by knockdown of the P2Y_2_ receptor (Supplementary Figure S1D,E) or by treatment with the P2Y inhibitor, suramin (Figure S1F). This increase was also inhibited by the PLC inhibitor, U73122 (Supplementary Figure S1F). To determine the source of the intracellular Ca^2+^, the effect of ATP, UTP or ATPγS was assessed in the absence of extracellular Ca^2+^. Removal of extracellular Ca^2+^ did not affect the increase in [Ca^2+^]_i_ (Figure S1G). However, this increase was blocked by pre-incubation with thapsigargin, which depletes intracellular Ca^2+^ stores (Figure S1G), indicating that ATP provoked Ca^2+^ release from the SR. Although other subtypes of P2Y and P2X receptors are known to be expressed in C2C12 cells [24], the facts that removal of extracellular Ca^2+^ had no effect on the increase in [Ca^2+^]_i_ (Supplementary Figure S1G), and that knockdown of the P2Y_2_ receptor almost completely inhibited the ATP-induced increase in [Ca^2+^]_i_ (Figure S1E), suggest that other purinergic receptors were not involved in ATP- or UTP-induced increase in [Ca^2+^]_i_ in vitro. Furthermore, the increase in [Ca^2+^]_i_ was inhibited either by the overexpression of an IP_3_-sponge (Figure S1H), a shortened and soluble form of the IP_3_R containing its high-affinity IP_3_-binding sequence [26,27], or by the IP_3_R inhibitor, xestospongin C (XeC) (Figure S1I). These results indicate that the ATP-induced increase in [Ca^2+^]_i_ occurs by activating the P2Y_2_ receptor and the downstream PLC/IP_3_R pathway in C2C12 myotubes.

### 2.2. ATP Activates mTOR via the P2Y_2_ Receptor/PLC/IP_3_R Pathway In Vitro

We have previously shown that the TRPV1-mediated increase of [Ca^2+^]_i_ activates mTOR, and promotes muscle hypertrophy [8,9]. Therefore, to investigate the downstream events of the ATP-induced increase in [Ca^2+^]_i_, we focused on the activation of mTOR. Treatment of C2C12 myotubes with ATP or UTP induced phosphorylation of p70S6K at Thr389, which is an mTOR-regulated phosphorylation site indispensable for p70S6K activation [28], in a concentration-dependent manner (Figure 2C,D). Furthermore, this phosphorylation was inhibited by the mTOR inhibitor, rapamycin (Figure 2E), indicating that mTOR was activated by ATP. It is worth noting that phosphorylation of Akt was not altered by ATP or UTP (Figure 2C). Nevertheless, phosphorylation of p70S6K was prevented by the PI3K inhibitor, LY294002 (Figure 2E), suggesting an involvement of PI3K in the activation of mTOR. Because LY294002 inhibits both, class I and class III PI3K [29], we investigated which PI3K was involved in the activation of mTOR by gene knockdown of Vps34, a member of class III PI3K, as well as p85, a member of class I PI3K [30]. Knockdown of Vps34 completely prevented the ATP- or UTP-induced phosphorylation of p70S6K (Figure 2F), whereas knockdown of p85 had no effect (Figure 2G). These results indicate that the ATP-induced activation of mTOR was mediated by class III PI3K, rather than class I PI3K/Akt, the major downstream pathway of IGF-1.

**Figure 3:**
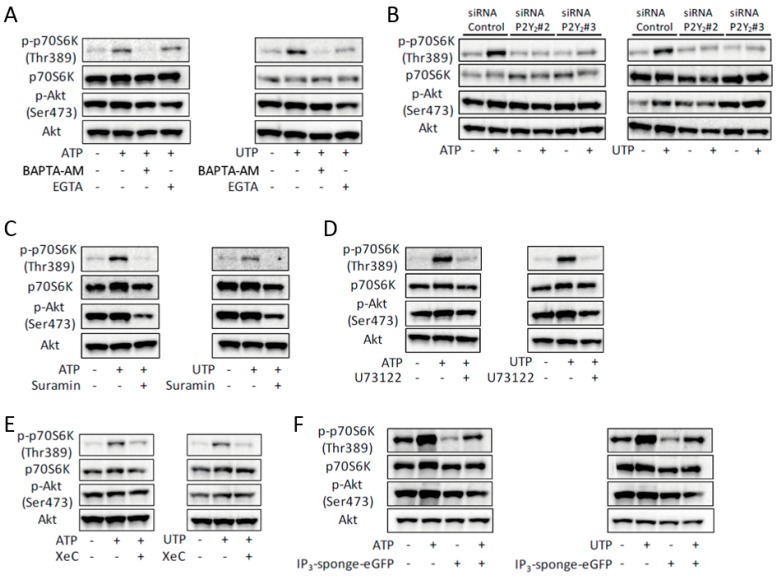
ATP-induced activation of mTOR is mediated by the P2Y_2_ receptor and its downstream PLC/IP_3_R pathway in C2C12 myotubes. (**A**–**E**) Representative Western blots showing the effects of BAPTA-AM or EGTA (**A**), siRNA for the P2Y_2_ receptor (**B**), suramin (**C**), U73122 (**D**), XeC (**E**) on ATP- or UTP-induced phosphorylation of p70S6K and Akt (*n* = 4). (**F**) ATP- or UTP-induced phosphorylation of p70S6K and Akt in the IP_3_-sponge-overexpressed C2C12 myotubes (*n* = 4). The concentration of ATP and UTP was 100 µM and the duration of treatment in all experiments shown in this figure was 30 min.

The ATP- or UTP-induced phosphorylation of p70S6K was prevented by the intracellular Ca^2+^ chelator, BAPTA-AM, indicating that the activation of mTOR was [Ca^2+^]_i_-dependent (Figure 3A). Removal of extracellular Ca^2+^ by EGTA partially prevented phosphorylation of p70S6K (Figure 3A). In line with the effects on the ATP- or UTP-induced increase of [Ca^2+^]_i_ (Figure S1), knockdown of P2Y_2_ prevented the ATP- or UTP-induced phosphorylation of p70S6K (Figure 3B). Treatment with suramin, U73122, XeC, or overexpression of IP_3_-sponge-eGFP, also prevented this phosphorylation (Figure 3C–F). Taken together, these results indicate that ATP induced activation of mTOR, and that this activation is mediated by the P2Y_2_ receptor/PLC/IP_3_R pathway.

### 2.3. ATP Upregulates JunB and IL-6 by Activating MAPKs and mTOR

**Figure 4:**
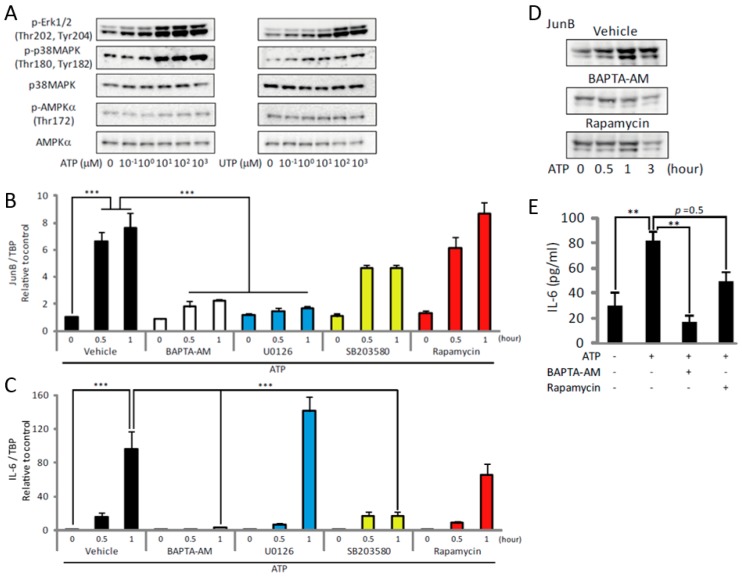
ATP upregulates JunB and IL-6 by activating Erk1/2, p38 MAPK and mTOR in C2C12 myotubes. (**A**) Concentration-dependent effects of ATP (left) or UTP (right) on phosphorylation of Erk1/2, p38 MAPK and AMPKα (*n* = 4). (**B**,**C**) Gene expression analysis showing the effects of BAPTA-AM (white), U0126 (blue), SB203580 (yellow) and rapamycin (red) on the ATP-induced expression of JunB (**B**) and IL-6 (**C**) (*n* = 4). (**D**) Representative Western blots showing the effects of BAPTA-AM or rapamycin on the ATP-induced increase of JunB (*n* = 4). (**E**) Effects of BAPTA-AM or rapamycin on the ATP-induced increase of IL-6 protein level (*n* = 4). The concentration of ATP in (**B**–**E**) was 100 µM and the duration of treatment in all experiments shown in this figure was 30 min. ** *p* < 0.01, *** *p* < 0.001 by ANOVA with Tukey-Kramer test. Error bars indicate S.E.M.

Treatment with ATP or UTP also induced phosphorylation of Erk1/2 and p38 mitogen-activated protein kinases (MAPK), whereas the degree of phosphorylation of AMP-activated protein kinase α (AMPKα) remained unchanged (Figure 4A). These results indicate that the ATP-induced increase in [Ca^2+^]_i_ activated both mTOR and MAPKs, suggesting a coordinated effect on subsequent events. To identify the potential downstream targets of Erk1/2 and p38 MAPK, we focused on JunB and interleukin-6 (IL-6), which are known to be regulated by Erk1/2 and p38 MAPK. These genes are upregulated by exercise or mechanical load, and are important for subsequent muscle hypertrophy [31,32,33,34,35,36,37]. Exposure of C2C12 myotubes to ATP increased the expression of JunB and IL-6 (Figure 4B,C, black), but this was prevented by BAPTA-AM (Figure 4B,C, white), indicating that their expression is [Ca^2+^]_i_-dependent. Treatment of the myotubes with the MEK inhibitor, U0126, blocked the upregulation of JunB by ATP, whereas treatment with the p38 MAPK inhibitor, SB203580, prevented the upregulation of IL-6 (Figure 4B,C, blue and yellow). These results indicate that ATP induced the transcription of JunB and IL-6 through the [Ca^2+^]_i_-dependent activation of Erk1/2 and p38 MAPK pathways, respectively. Treatment with rapamycin had no effect on the upregulation of JunB or IL-6 (Figure 4B,C, red). However, the ATP- or UTP-induced increase of JunB or IL-6 at the protein level was prevented by rapamycin (Figure 4D,E), suggesting that the ATP/UTP-induced activation of Erk1/2 and p38 MAPK contributed to the transcription of JunB and IL-6, and that the activation of mTOR promoted their translation.

### 2.4. ATP Induces an Increase in [Ca^2+^]_i_ Specifically in Slow Muscles

**Figure 5:**
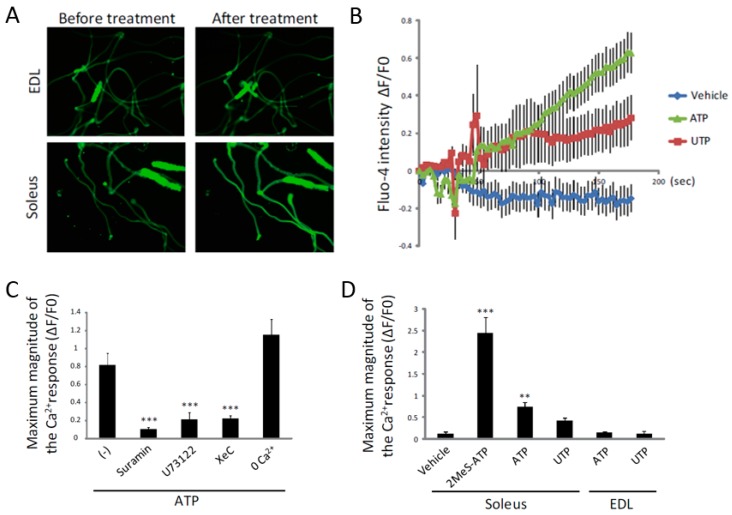
ATP induces an increase in [Ca^2+^]_i_ by the activation of the P2Y receptor and its downstream PLC/IP_3_R pathway in single fibers isolated from soleus muscle. (**A**) Representative fluorescence imaging of Fluo-4-loaded single fibers isolated from EDL (upper) and soleus (lower) muscles before and after treatment with ATP for 3 min. (**B**) Traces of Fluo-4 fluorescence in single fibers isolated from soleus muscle. (**C**) Quantitative analysis for the maximum magnitudes of Fluo-4 intensity in suramin-, U73122-, XeC- or 0 Ca^2+^-buffer-treated single fibers isolated from soleus muscle. (**D**) Quantitative analysis of the maximum Fluo-4 intensity in 2MeS-ATP-, ATP- or UTP-treated soleus or EDL single fibers at a concentration of 100 µM. At least 10 fibers were analyzed in B, C and D. ** *p* < 0.01, *** *p* < 0.001 by ANOVA with Tukey-Kramer test. Error bars indicate S.E.M.

To investigate the effects of ATP on mature muscle fibers, we isolated single fibers from the soleus and extensor digitorum longus (EDL) muscles. Similarly to the results of a previous report [38], ATP had no effect on [Ca^2+^]_i_ in EDL fibers (Figure 5A). However, treatment with ATP showed a slow, but significant increase in [Ca^2+^]_i_ in the soleus fibers, suggesting that ATP increases [Ca^2+^]_i_ only in slow fibers (Figure 5A,B). Similarly to C2C12 myotubes, the ATP-induced increase of [Ca^2+^]_i_ in soleus fibers was inhibited by suramin, U73122 and XeC, but not by removal of extracellular Ca^2+^ (Figure 5C), suggesting that the effect of ATP on single fibers was also mediated by the P2Y receptor and the downstream PLC/IP_3_R pathway. However, different to C2C12 myotubes, the effects of 2MeS-ATP and UTP on [Ca^2+^]_i_ were significantly higher and lower than that of ATP, respectively (Figure 5D). These results suggest that the target P2Y receptor of ATP might not be the P2Y_2_ receptor in mature muscle fibers.

### 2.5. ATP Induces Muscle Hypertrophy by Regulating [Ca^2+^]_i_

**Figure 6:**
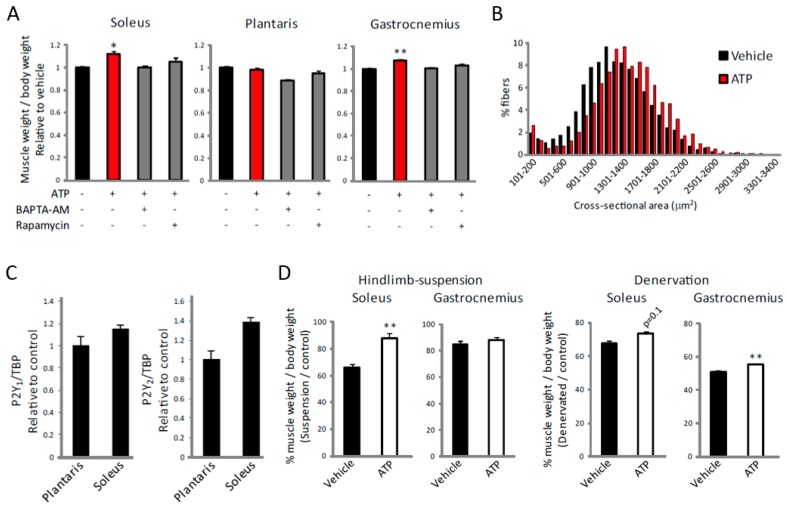
ATP induces muscle hypertrophy and alleviates muscle atrophy in an [Ca^2+^]_i_- and mTOR-dependent manner. (**A**) Effects of ATP with or without BAPTA-AM or rapamycin on the increase of muscle weight in soleus, plantaris and gastrocnemius muscles (*n* = 4). (**B**) The cross-sectional area distributions were plotted as frequency histograms. At least 2800 fibers from 4 different soleus muscles were counted. (**C**) Expression of P2Y_1_ and P2Y_2_ receptor mRNA in soleus and plantaris muscle (*n* = 8). (**D**) Effects of ATP on soleus and gastrocnemius muscles weights in hindlimb-suspended (left) or denervated (right) muscle shown as a percentage of the control group (*n* > 4). * *p* < 0.05, ** *p* < 0.01 by ANOVA with Tukey-Kramer test in (**A**), and ** *p* < 0.01 by Student’s *t*-test in (**D**). Error bars indicate S.E.M.

Finally, to investigate the effect of the ATP-induced increase in [Ca^2+^]_i_ on muscle hypertrophy in vivo, ATP was administered intramuscularly daily for 1 week. This treatment led to a slight but significant increase in muscle weight in both the soleus and gastrocnemius muscles (Figure 6A). Administration of ATP also increased fiber size in the soleus muscle (Figure 6B). Consistent with the slow fiber-specific effect of ATP (Figure 5), this treatment had no effect on plantaris muscle (Figure 6A), even though we did not observe significant differences in the expression of P2Y_1_ or P2Y_2_ receptors between soleus and plantaris muscles (Figure 6C). This increase was not observed when BAPTA-AM or rapamycin was co-administered (Figure 6A), indicating that ATP promotes muscle hypertrophy by regulating [Ca^2+^]_i_ and mTOR. Furthermore, administration of ATP during hindlimb-suspension or denervation attenuated the decrease of muscle weight (Figure 6D). Taken together, these results suggest the P2Y receptor to be a potential therapeutic target for treating muscle atrophy.

## 3. Discussion

Extracellular ATP plays a crucial role in the regulation of a considerable number of biological processes [39,40]. Plasma ATP levels are increased by exercise or muscle contraction and can function in an autocrine or paracrine manner [10,11,12,13]. Of note, an earlier study showed that ATP was released from skeletal muscle in response to electrical stimulation through the pannexin channel, suggesting a regulated involvement of extracellular ATP in contraction- or exercise-induced events [39]. However, to our knowledge, the role of extracellular ATP on the induction of muscle hypertrophy has not been studied. Therefore, we investigated the ATP-dependent activation of the P2Y receptor and its downstream PLC/IP_3_R pathway in the context of muscle hypertrophy. ATP activated the P2Y_2_ receptor and initiated a subsequent PLC-mediated increase in [Ca^2+^]_i_ through IP_3_R in vitro. In turn, this led to the activation of mTOR and MAPKs, and subsequent expression of JunB or IL-6. Furthermore, ATP affected [Ca^2+^]_i_ and muscle hypertrophy in soleus muscle, but not in EDL or plantaris muscles, suggesting that ATP regulates [Ca^2+^]_i_ specifically in slow muscles. This is the first report that shows the role of extracellular ATP in the activation of mTOR and subsequent muscle hypertrophy. Our discovery demonstrates an important role of extracellular ATP and intracellular Ca^2+^ signaling in transducing physical activity into activation of intracellular signaling pathways that subsequently lead to muscle hypertrophy.

The IGF-1-induced activation of Akt, which is mediated by class I PI3K, is essential for inducing muscle growth and hypertrophy [2]. Although the regulation of Akt by Ca^2+^ has been reported [41,42], our results show that the effect of [Ca^2+^]_i_ on mTOR was not mediated by class I PI3K or Akt (Figure 2G), suggesting that the IGF-1-induced muscle growth and the exercise-induced muscle hypertrophy are differently regulated. Consistent with these results, Gulati et al. demonstrated that amino acid-induced activation of mTOR was mediated by increased [Ca^2+^]_i_, and that the direct target of Ca^2+^ was hVps34, a component of class III PI3K in Hela cells [43]. Vps34 regulates intracellular protein trafficking and autophagy, and is known to be activated by a variety of physiological stimuli, such as exercise [44,45]. These results suggest that both, amino acid- and ATP-induced activation of mTOR share similar mechanisms that are mediated by [Ca^2+^]_i_ and Vps34. However, in contrast to our observation, Mercan et al. showed that leucine-induced increase of [Ca^2+^]_i_ and subsequent activation of mTOR was not mediated by hVps34 in C2C12 myoblasts [46]. Currently, the differences between Vps34-dependent and –independent activation of mTOR are still unclear. Future studies using adequate experimental models are required in order to elucidate the molecular links between [Ca^2+^]_i_ and mTOR as well as the role of mTOR phosphorylation in response to ATP.

Moreover, we showed JunB and IL-6 to be downstream targets of ATP-induced activation of MAPKs. Several lines of evidence suggest that the exercise-induced upregulation of IL-6 is controlled by [Ca^2+^]_i_ [31,47]. Our results indicate that this also applies to JunB. Raffaello et al. showed that overexpression of JunB was sufficient to increase protein synthesis and muscle hypertrophy without activating the Akt/mTOR pathway [33]. In view of our observation that the ATP-induced increase of JunB at the protein level was inhibited by rapamycin (Figure 4D), the increase of protein synthesis after physiological stimulation, which is considered to be due solely to mTOR, might be at least partially caused by JunB that was upregulated by mTOR.

Most of our results regarding signal transduction in C2C12 myotubes are similar to those in mature muscle fibers. However, it appears that the receptor activated by ATP was different in C2C12 myotubes as compared to mature muscle. Because the relative agonist potencies for the P2Y_1_ receptor are 2MeS-ATP > ATP [48], we suspected the potential involvement of P2Y_1_, or a combination of P2Y_1_ and other P2Y receptors in the increase of [Ca^2+^]_i_ in soleus muscle fibers. Furthermore, and interestingly, the effects of ATP in vivo appeared to occur only in slow muscles, even though we did not detect a difference in expression levels of P2Y_1_ and P2Y_2_ receptor between fast and slow muscles (Figure 6C). A previous study showed that the expression level of IP_3_R was much higher in slow muscle than in fast muscle [49], suggesting that the different levels of IP_3_R, or a post-transcriptional regulation of P2Y receptors might be involved in the different responses to ATP between fast and slow fibers. Notably, our results do not exclude an involvement of other proteins, such as ATP transporters or pannexin channels in the exercise-induced events.

The concentration of ATP in the blood is thought to be kept below 1 µM [12]. Our results indicate that more than 1 µM of ATP was required to activate mTOR (Figure 2C). It is possible that ATPase activity keeps overall circulating ATP low, but cell-adjacent concentrations of ATP might be much higher than 1 µM. If this is the case, a physiological role for exercise-induced increase of extracellular ATP, activation of mTOR and subsequent muscle hypertrophy would exist. Furthermore, although the effect of ATP on muscle in vivo might be below what we observed in this study, it is likely that the repeated increase of extracellular ATP levels occurring in contracting muscle contributes to activate mTOR and muscle hypertrophy. Our results on ATP administration to mice showing an increase in soleus muscle weight (Figure 5 and Figure 6) are in favor of such a role, at least for slow muscle. In addition, although the exercise-induced increase of [Ca^2+^]_i_ returned to a steady-state level within 1 h (Figure 1B), it is likely that the ATP-induced signaling cascade persists for a much longer period after exercise. In addition to the target P2Y receptor subtype of ATP in vivo, its role in the exercise-induced activation of mTOR and subsequent muscle hypertrophy should be analyzed in more detail in the future.

We have previously shown that mechanical load activates neuronal nitric oxide synthase (nNOS) and promotes muscle hypertrophy by regulating TRPV1 [8]. In the current study, the ATP-induced increase in [Ca^2+^]_i_ occurs exclusively in slow muscle (Figure 5 and Figure 6). It is noteworthy that NOS activity is much greater in fast than slow muscle [50]. Considering that both plantaris and soleus muscle showed increase in [Ca^2+^]_i_ after exercise (Figure 1), the subsequent activation of mTOR might be differently regulated in different fiber types.

Finally, our results point to the P2Y receptor as a therapeutic target for the treatment of muscle atrophy, as it occurs in immobilized muscle or in cancer cachexia. The anti-cancer activity of purine-based agonists has already been demonstrated. In fact, intraperitoneal injection of ATP has been shown to inhibit cachexia and prolong survival in mice [51]. Although the administered ATP was thought to alleviate cachexia by inhibiting tumor growth [51], our results suggest that part of its effect may be mediated by an action on skeletal muscle. In conclusion, our results indicate that the ATP-induced activation of the P2Y receptor and the subsequent increase in [Ca^2+^]_i_ through IP_3_R convert a mechanical load into activation of intracellular signaling pathways, subsequently leading to muscle hypertrophy.

## 4. Materials and Methods

### 4.1. Animals

Twelve to 14-week-old male C57BL/6 mice were purchased from Nihon CREA. pCAGGS-GCaMP2 mice were provided by Dr. Michael Kotlikoff [17]. All mice were housed at the animal facility at the National Center of Neurology and Psychiatry (NCNP, Tokyo, Japan). All of the animal procedures were approved by the Experimental Animal Care and Use Committee at the National Center of Neurology and Psychiatry (Approval number: 2016001, Approval date: 2/25/2016). All of the experimental methods were performed in accordance with approved guidelines.

### 4.2. Materials

Antibodies against Akt (#9272), phospho-Akt (Ser473) (#9271), phospho-Akt (Thr308) (#9275), p70S6K (#9202), phospho-p70S6K (Thr389) (#9205), p85 (#4257), Vps34 (#3358), AMPKα (#2603), phospho-AMPKα (Thr172) (#2535), phospho-ERK (Thr202/Tyr204) (#9101), p38 MAPK (#9212), and phospho-p38 MAPK (Thr180, Tyr182) (#9211) were purchased from Cell Signaling Technology. The P2Y_2_ antibody (ab10270) came from Abcam. The JunB (sc-46) and α-tubulin (sc-12462) antibodies were obtained from Santa Cruz. The laminin α2 (Clone: 4H-8, ALX-804-190) antibody was supplied by Alexis Biochemicals (San Diego, CA, USA). The following compounds were obtained from the sources indicated and used at the final concentrations indicated: ATP (0.1–1000 µM, Sigma-Aldrich, Saint Louis, MO, USA), UTP (0.1–1000 µM, Sigma-Aldrich), BAPTA-AM (50 µM, Calbiochem, Sigma-Aldrich, Saint Louis, MO, USA), EGTA (2 mM, Sigma-Aldrich), thapsigargin (2 µM, Calbiochem), rapamycin (0.1 µM, Sigma-Aldrich), LY294002 (10 µM, Sigma-Aldrich), suramin (100 µM, Sigma-Aldrich), U73122 (10 µM, Sigma-Aldrich), XeC (2 µM, Sigma-Aldrich), GTP (100 µM, Sigma-Aldrich), CTP (100 µM, Sigma-Aldrich), TTP (100 µM, Sigma-Aldrich), ATPγS (100 µM, Sigma-Aldrich), 2MeS-ATP (100 µM, Sigma-Aldrich), ADP (100 µM, Sigma-Aldrich), AMP (100 µM, Sigma-Aldrich), adenosine (100 µM, Sigma-Aldrich), U0126 (10 µM, Sigma-Aldrich) and SB203580 (10 µM, Sigma-Aldrich).

### 4.3. C2C12 Cell Culture

C2C12 myoblasts were cultured in a growth medium (DMEM; (Wako) supplemented with 10% FBS and 1% penicillin-streptomycin) at 37 °C with 5% CO_2_ as previously described [52]. Myogenic differentiation was induced by changing the growth medium to a differentiation medium (DMEM supplemented with 2% horse serum and 1% penicillin-streptomycin) at 37 °C with 5% CO_2_. For Western blotting, myotubes were deprived of serum for 8 h, and then cultured for an additional 2 h in Earle’s Balanced Salt Solution (Sigma-Aldrich) to deprive both serum and amino acids. C2C12 myotubes were treated with the indicated reagents for 30 min.

### 4.4. Gene Knockdown by RNA Interference

The transfection of siRNA (100 nM, Sigma-Aldrich) into C2C12 myotubes was performed using jetPRIME (Polyplus). The following siRNA sequences were used: p85#1 5′-ggaauaugauagauuauautt-3′, p85#3 5′-ggauuaugcauaaccaugatt-3′, Vps34#2 5′-caucugaccacgaucucaatt-3′, Vps34#3 5′-cuacaaggcguuuaguacatt-3′, P2Y_2_#1 5′-caaucauuuguacgugugatt-3′, P2Y_2_#2 5′-cuaaggacauucggcuauatt-3′, P2Y_2_#3 5′-gucuugacccgguacucuatt-3′. The control siRNA was purchased from Sigma-Aldrich.

### 4.5. Retrovirus-Mediated Gene Transfer

The red genetically encoded Ca^2+^ indicators for optimal imaging (R-GECO) [53] or the IP_3_-sponge-eGFP cDNA were cloned into a pMXs-puro vector [54]. Viral particles were prepared by introducing the resultant pMXs-R-GECO and pMXs-IP_3_-sponge-eGFP into PLAT-E retrovirus packaging cells using polyethylenimine (Polysciences, Warrington, PA, USA) [55]. Subsequently, the concentrated supernatant was added to the C2C12 myoblast culture containing 8 ng/mL polybrene (Sigma-Aldrich). The next day, cells stably expressing the viral vector were selected with puromycin (Invitrogen, Carlsbad, CA, USA).

### 4.6. Intracellular Ca^2+^ Level Measurement

For in vitro experiments, [Ca^2+^]_i_ was monitored using Fluo-4 or R-GECO as previously described [8]. Briefly, C2C12 myotubes were cultured in a serum-free medium for 6 to 9 h and then cultured for an additional 2 h in a buffer containing 140 mM NaCl, 5 mM KCl, 2.5 mM CaCl_2_, 1 mM MgCl_2_, 10 mM HEPES and 10 mM glucose (pH 7.0). Single fibers from soleus or EDL muscles were isolated by digestion using type 1 collagenase as previously described [9,52]. Myotubes or isolated single fibers were incubated in 4 µM Fluo-4 AM (Dojindo, Rockville, MA, USA) for 30 min at room temperature to allow homogenous intracellular distribution of the dye. After incubation at 37 °C, the Fluo-4 loaded cells were placed on the stage of an inverted microscope (Olympus, Tokyo, Japan), and fluorescent intensity changes were recorded every 3 s. Data were calculated as normalized fluorescence ΔF/F0: ΔF/F0 = (Fmax − F0)/F0, where Fmax was the maximum fluorescence and F0 was the fluorescence before exposure to a reagent. Where noted, thapsigargin was combined with Fluo-4 AM, and the extracellular Ca^2+^ was depleted using a Ca^2+^-free buffer containing 2 mM EGTA. For in vivo Ca^2+^ imaging analysis, soleus or plantaris muscles were immediately dissected from pCAGGS-GCaMP2 mice before or after exercise, and the fluorescence was observed using a Biozero digital microscope (Keyence, Osaka, Japan).

### 4.7. Western Blot Analysis

Western blot analysis was performed as described previously [52] with minor modifications. Briefly, cells were frozen immediately by liquid nitrogen, and protein was extracted using a sample buffer containing 0.1% Triton X-100, 50 mM HEPES (pH 7.4), 4 mM EGTA, 10 mM EDTA, 15 mM Na_4_P_2_O_7_, 100 mM glycerophosphate, 25 mM NaF, 5 mM Na_2_VO_4_ and a complete protease inhibitor cocktail (Roche, Basel, Switzerland). Protein concentrations were determined by Coomassie Brilliant Blue G-250 (Bio-Rad, Hercules, CA, USA). Just before SDS-PAGE, an aliquot of the extracted protein solution was mixed with an equal volume of sample loading buffer containing 30% glycerol, 5% 2-mercaptoethanol, 2.3% SDS, 62.5 mM Tris-HCl (pH 6.8) and 0.05% bromophenol blue. The mixture was heated at 60 °C for 10 min. Proteins were separated on an SDS-polyacrylamide gel and electrically transferred from the gel to a polyvinylidene difluoride membrane (Millipore, Burlington, MA, USA). The signals were detected using the ECLTM Western Blotting Detection system and ImageQant LAS 4000 (GE Healthcare, Chicago, IL, USA), quantified by scanning densitometry (ImageQuant TL) and expressed in arbitrary units.

### 4.8. RNA Preparation and RT-qPCR

RT-qPCR was performed as described previously [52] with minor modifications. Briefly, total RNA was isolated by TRIzol (Invitrogen). Single strand cDNA was synthesized by the QuantiTect Reverse Transcription Kit (Qiagen, Hilden, Germany). Expression levels of JunB, IL-6, P2Y_1_ and P2Y_2_ receptors were evaluated by quantitative RT-PCR using SYBR Premix Ex Taq II (Takara, Kyoto, Japan) on a MyiQ single-color system (Bio-Rad), and were normalized to the TATA-binding protein (TBP). Primer sequences for RT-qPCR were as follows: JunB forward: 5′-ctcaacctggcggatccctatc-3′, reverse: 5′-gtgtctgatccctgacccgaaa-3′; IL-6 forward: 5′-agatctactcggcaaacc-3′, reverse: 5′-cgtagagaacaacataagtcag-3′; P2Y_1_ forward: 5′-cgtggctatctggatgttcgt-3′, reverse: 5′-agatgagggctggtagggt-3′; P2Y_2_ forward: 5′-ctgggatacaagtgtcgtt-3′, reverse: 5′-atagagagccacgacgtt-3′; TBP forward: 5′-cagcctcagtacagcaatcaac-3′, reverse: 5′-taggggtcataggagtcattgg-3′.

### 4.9. Exercise Model

Exercise experiment was performed as described previously [8,9] with minor modifications. Mice were placed on a flat MK-680S treadmill (Muromachi Kikai, Tokyo, Japan) and forced to run at 5 m/min. After 5 min, the speed was gradually increased by 1 m/min every minute. After 30 min, the test was stopped, and gastrocnemius or plantaris muscle were immediately isolated.

### 4.10. ELISA

The release of IL-6 elicited by ATP (100 µM) from C2C12 myotubes was measured in both, the absence and the presence of BAPTA-AM (50 µM) or rapamycin (10 µM). Myotubes were deprived of serum for 8 h, and then cultured for an additional 2 h in Earle’s Balanced Salt Solution to deprive both serum and amino acids. Four hours after stimulation by ATP, 50 µL samples were taken from each culture plate and IL-6 levels were measured using an ELISA kit (Thermo Scientific, Waltham, MA, USA).

### 4.11. Hindlimb-Suspension and Denervation Surgery

Hindlimb-suspension analysis and denervation surgery were performed as described previously [8]. For hindlimb-suspension analysis, mice were randomly assigned to control or hindlimb-suspension groups. To induce muscle atrophy, mice were suspended by their tail so that their hindlimbs were 2 mm off the floor for 14 days. For denervation surgery, the sciatic nerve was excised from a small incision made in the mid-lateral thigh under general anesthesia. Mice were sacrificed 14 days after surgery. Muscle weight was normalized to body weight and was presented as a percentage of the control group. ATP (500 µM; 150 µL) was injected into hindlimb muscles once a day for 13 days. Since the injected volume was 150 µL, and muscle weights were about 200–300 mg, a dilution of more than two-fold should result.

### 4.12. Histological and Immunohistological Analysis

Histological and immunohistological analysis were performed as described previously [52,56,57] with minor modifications. After cervical dislocation, soleus muscle was immediately isolated and frozen in isopentane cooled by liquid nitrogen. Eight-µm thick cryosections were cut across the middle part of the muscle. For immunohistochemistry, cryosections were fixed in cold acetone for 10 min, and incubated in PBS containing 1% bovine serum albumin and 5% goat serum for 15 min at room temperature. Anti-laminin-α2 antibody in PBS containing 1% bovine serum albumin was applied overnight at 4 °C. Following incubation with secondary antibodies, mounted sections were observed using a Biozero digital microscope (Keyence), and cross-sectional areas were determined.

### 4.13. Statistical Analysis

All values are expressed as mean ± S.E.M. Statistical differences were assessed by the Student’s *t*-test or one-way analysis of variance, with differences among the groups assessed by a Tukey-Kramer post-hoc analysis. Probabilities less than 5% (*, *p* < 0.05), 1% (**, *p* < 0.01) or 0.1% (***, *p* < 0.001) were considered to be statistically significant.

## Supplementary Materials

Supplementary materials can be found at http://www.mdpi.com/1422-0067/19/9/2804/s1.

## Abbreviations

AMPKα	AMP-activated protein kinase α
EDL	extensor digitorum longus
IP_3_R	inositol 1,4,5-trisphosphate receptor
IGF-1	linear dichroism
IL-6	interleukin-6
[Ca^2+^]_i_	intracellular Ca^2+^ level
mTOR	mammalian target of rapamycin
MAPK	mitogen-activated protein kinase
nNOS	neuronal nitric oxide synthase
PI3K	phosphatidylinositol 3-kinase
PLC	phospholipase C
SR	sarcoplasmic reticulum
TBP	TATA-binding protein
XeC	xestospongin C
