# Supplementary material for: ATP-Induced Increase in Intracellular Calcium Levels and Subsequent Activation of mTOR as Regulators of Skeletal Muscle Hypertrophy
Source: Int J Mol Sci. 2018 Sep 18;19(9):2804. doi: 10.3390/ijms19092804 (PMC6163678; doi:10.3390/ijms19092804)
Supplement: Supplementary file 1 [file ijms-19-02804-s001.pdf]

ATP-induced increase in intracellular calcium levels and subsequent activation of mTOR as regulators of skeletal muscle hypertrophy

Naoki Ito, Urs T. Ruegg and Shin'ichi Takeda

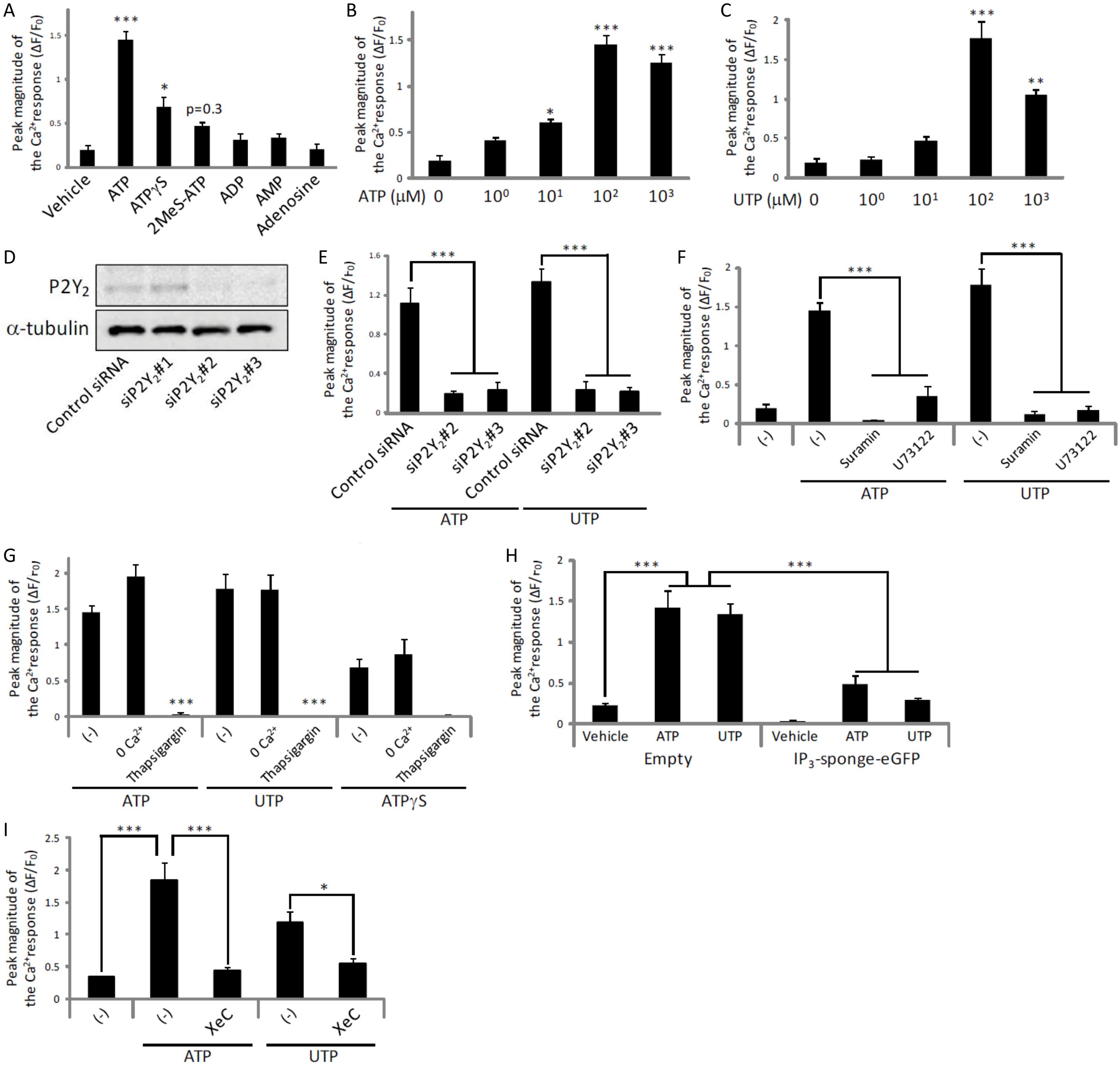

Supplementary figure 1. ATP induces increase in $[Ca^{2+}]_i$ by activation of the $P2Y_2$ receptor/PLC/IP_3R pathway in C2C12 myotubes related to Figure 2. (**A**) Quantitative analysis of the peak magnitudes of Fluo-4 intensity in ATP or ATP analog-treated C2C12 myotubes ($n = 4$). (**B** and **C**) Concentration-dependent effects of ATP (B) or UTP (C) on the peak magnitude of the Ca^{2+} response ($n = 4$). (**D**) Western blot analysis of the $P2Y_2$ receptor in siRNA-transfected C2C12 myotubes. (**E**) Quantitative analysis showing the effect of $P2Y_2$ gene knockdown on ATP- or UTP-induced increases in $[Ca^{2+}]_i$ ($n = 4$). (**F**) Quantitative analysis for the peak magnitudes of Fluo-4 intensity in suramin- or U73122-treated C2C12 myotubes with ATP or UTP (100 μM) ($n = 4$). (**G**) Quantitative analysis of the peak magnitudes of Fluo-4 intensity in thapsigargin-treated C2C12 myotubes, or C2C12 myotubes in 0 Ca^{2+} buffer with ATP, UTP or ATPγS (100 μM) ($n = 4$). (**H**) Quantitative analysis showing the effect of overexpression of an IP_3-sponge-eGFP on ATP- or UTP-induced increases of $[Ca^{2+}]_i$ ($n = 4$). (**I**) Quantitative analysis of the peak magnitudes of Fluo-4 intensity in XeC-treated C2C12 myotubes ($n = 4$). *$P < 0.05$, **$P < 0.01$, ***$P < 0.001$ by ANOVA with Tukey-Kramer test. Error bars indicate S.E.M.
